# Validity of predictive equations for total energy expenditure against doubly labeled water

**DOI:** 10.1038/s41598-024-66767-7

**Published:** 2024-07-08

**Authors:** Olalla Prado-Nóvoa, Kristen R. Howard, Eleni Laskaridou, Guillermo Zorrilla-Revilla, Glen R. Reid, Elaina L. Marinik, Brenda M. Davy, Marina Stamatiou, Catherine Hambly, John R. Speakman, Kevin P. Davy

**Affiliations:** 1 Department of Human Nutrition, Foods, and Exercise, Human Integrative Physiology Laboratory, Virginia Tech, Blacksburg, VA USA; 2 Graduate Program in Translational Biology, Medicine, and Health, Virginia Tech, Blacksburg, VA USA; 3 Laboratorio de Evolución Humana, Departamento de Historia, Geografía y Comunicación, Universidad de Burgos, Burgos, Spain; 4 CIAS–Research Centre for Anthropology and Health, University of Coimbra, 3020 Coimbra, Portugal; 5 Institute of Biological and Environmental Sciences, University of Aberdeen, Aberdeen, AB24 2TZ Scotland, UK; 6 Shenzhen Key Laboratory of Metabolic Health, Center for Energy Metabolism and Reproduction, Shenzhen Institutes of Advanced Technology, Chinese Academy of Sciences, Shenzhen, China

**Keywords:** Total energy expenditure, Predictive equations, Physical activity, Doubly labeled water, Metabolism, Translational research

## Abstract

Variations in physical activity energy expenditure can make accurate prediction of total energy expenditure (TEE) challenging. The purpose of the present study was to determine the accuracy of available equations to predict TEE in individuals varying in physical activity (PA) levels. TEE was measured by DLW in 56 adults varying in PA levels which were monitored by accelerometry. Ten different models were used to predict TEE and their accuracy and precision were evaluated, considering the effect of sex and PA. The models generally underestimated the TEE in this population. An equation published by Plucker was the most accurate in predicting the TEE in our entire sample. The Pontzer and Vinken models were the most accurate for those with lower PA levels. Despite the levels of accuracy of some equations, there were sizable errors (low precision) at an individual level. Future studies are needed to develop and validate these equations.

## Introduction

Energy requirements of non-reproductive adults are defined as the amount of energy from food needed to balance energy expenditure so as to maintain body mass and composition as well as to meet the needs to maintain a level of physical activity associated with long-term health^1^. When body mass is stable, energy requirements are equivalent to total energy expenditure (TEE)^1^. As such, accurate assessment of TEE is crucial to determine nutritional needs, but also to understand many physiological, biological, and evolutionary processes^2–4^. The doubly labeled water (DLW) technique is the gold standard for measuring free-living TEE^5^. However, the isotopes and their quantification are costly. As such, other approaches are needed when DLW is unavailable or unaffordable.

Physical activity energy expenditure (PAEE) is the most variable component of TEE^3,6^, making the estimation of daily requirements challenging. Several approaches have been used to quantify the cost of physical activity, including behavioral observation, questionnaires, heart rate, or motion sensors^7,8^, but these resulted in limited accuracy. The physical activity level (PAL) can also be calculated as the quotient of TEE and resting metabolic rate (RMR). In turn, TEE can be estimated as multiples of RMR. The latter is a practical approach for controlling for age, sex, body mass and composition as well as expressing energy requirements in a range of individuals varying in habitual physical activity^1^. The limitations of this approach have been discussed in previous studies^9,10^ and include multiplicative prediction errors of estimated RMR and that PAL violates the assumption of a non-zero intercept and assumes PAEE depends on the same factors influencing RMR. In a similar additive conception of TEE, others have added the estimated PAEE obtained from accelerometry to RMR^11,12^. However, this approach ignores sources of expenditure such as the thermic effect of food and thermoregulation costs, and was associated with considerable variability and limited accuracy^13^. More importantly, this approach assumes that expenditure on activity is additive to the cost at rest, and does not consider the possibility that TEE could be constrained^4,14,15^ or that compensation might occur^16,17^.

Several prediction equations have been developed in an attempt to provide accurate estimates of TEE^9,10,18^. Although limited in number^10^, body mass, body composition, sex, age, height, and other factors have been used to predict TEE^3,19–21^. Interestingly, the inclusion of PA from accelerometry in these predictive equations does not contribute significantly to the variability accounted for in TEE^10,14,21^. However, this brings the possibility of testing how different equations that include and exclude PA perform when applied to a sample of individuals with different objectively measured PAL and PAEE.

The purpose of the present study was to determine the accuracy and precision of the available equations to estimate TEE (see Table S1) compared to DLW outcomes in a sample of females and males varying in physical activity levels.

## Methods

### Participants

Fifty-six healthy individuals (20–58 years; 27 females) with a wide range of habitual physical activity levels were recruited as part of a larger study. The participants were uniformly distributed across levels of self-reported physical activity, walking and/or running from 0 km per week to more than 120 km per week^22^.

Exclusion criteria were applied to those who were smokers, pregnant or breastfeeding, following fad diets, taking medications that could influence TEE or its components, or with a medical history that prevented their participation in the study. The complete experimental study was approved by the Institutional Review Board at the Virginia Polytechnic Institute and State University (Virginia Tech) (IRB #21-567). All experiments were performed in accordance with relevant guidelines and regulations. The volunteers included in the study were properly informed and verbal and written consent were obtained. Detailed experimental procedures have been described previously^22^.

### Anthropometry, body composition, and resting metabolic rate (RMR)

Body mass (BM) (0.1 kg) and height (to the nearest cm) were obtained using a stand-on scale with stadiometer (Welch Allyn, Scale-Tronix 5002, Skaneateles Falls, NY, USA). Body mass index (BMI) was calculated as kg/m^2^. Body composition (Fat Mass, FM, and Fat-Free Mass, FFM) was measured by dual-energy X-ray absorptiometry (DXA scan, Lunar Digital Prodigy Advance, software enCORE version 15, GE Healthcare; Madison, WI, USA).

RMR was measured with indirect calorimetry (Parvo Medics, TrueOne 2400 Metabolic Measurement System, OUSW 4.3.4; Murray, Utah, USA) using a ventilated canopy in a rested state (after a minimum of 12 h with no exercise, and after fasting for 12 h) as described previously^22,23^. The last 30 min of a 45-min measurement period were used for analysis. RMR (kcal/day) was measured twice in an interval separated by 14 days. The second measurement of RMR was used for analysis after documenting stability of body mass and excellent test–retest reliability (r = 0.93; p < 0.001).

### Physical activity

Physical activity (PA) was assessed by self-report (walking/running km per week) and with a triaxial accelerometer (ActiGraph GT3X, Actigraph Corporation, Pensacola, FL). Participants wore the accelerometer around their waist continuously for 14 days, removing it only for swimming, showering/bathing, or sleeping. Data collection was described in Prado-Nóvoa et al.^22^. Only individuals with at least 4 days each week for at least 10 h a day or more wear time were included for analysis. Fifty-three individuals met the established wear time criteria. Mean vector magnitude counts per minute per day (VM CPM) on valid monitoring days were used to quantify physical activity levels objectively. Self-reported physical activity levels (in km/week) were correlated with mean daily steps (r = 0.72, p < 0.001) and VM CPM (r = 0.62, p < 0.001) measured with accelerometry.

### Total energy expenditure (TEE), physical activity energy expenditure (PAEE), and physical activity level (PAL)

TEE (kcal/day) was measured with Doubly Labeled Water (DLW) following standard procedures^24,25^. After the collection of a baseline urine specimen, the participants were orally dosed with deuterium (^2^H_2_) and oxygen-18 (^18^O) in the form of water (^2^H_2_^18^O). Doses were calculated according to each participant's body mass, with desired enrichment of 10% ^18^O and 5% ^2^H_2_, as follows (1):

1 $$\text{dose }\left(\text{ml}\right)=\frac{\text{Body mass }\left(\text{in g}\right) *\text{ desired excess enrichment}}{\text{dose enrichment}}$$

where desired excess enrichment = 618.923 body mass, kg^−0.305^; and dose enrichment (10%) 100,000 ppm^26^. Each participant was provided with a glass containing the precise dose required (weighed to 3 decimal points) and asked to consume all of the dose. To ensure that the entire dose of DLW was consumed, additional water was added to the dosing glass, which was also consumed. The time of dosing was recorded.

The second urine specimen was collected 3 h after the dose. Urine specimens at the second void of the day were then collected daily over 14 days and the timing of each sample collection recorded. Urine samples were encapsulated into capillaries and vacuum-distilled^27^. The resulting water was analyzed using a liquid water analyzer (Los Gatos Research^28^). Samples were run alongside three laboratory standards and three international standards (SLAP2; Standard Light Artic Precipitate, vSMOW2; Standard Mean Ocean Water, and GRESP; Greenland Summit Precipitation^26,29^) to correct for daily variation and convert delta values to parts per million. Isotope elimination rates were converted to TEE using Equation 1 from Speakman et al.^25^. After obtaining the TEE, PAEE (kcal/day) (2) and PAL (3) were calculated as follows^6^:

2 $$\text{PAEE}=\left(\text{TEE}\times 0.9\right)-\text{RMR}$$

3 $$\text{PAL}=\text{TEE}/\text{RMR}$$

### Prediction of TEE

TEE was predicted in our participants with 10 different models. We applied four equations published by Plucker et al.^20^, two equations published by Pontzer et al.^3^, and three equations published by Vinken et al.^19^. In addition, TEE was also estimated in our participants using the PAEE estimated by accelerometry, an assumed TEF (10%), and the measured RMR: RMR + ACC PAEE. A detailed description of all the models applied is provided in Supplementary Table 1. The models applied^3,19,20^ were selected because they are known predictive equations to estimate TEE based on individual characteristics not exclusively relying on an additive conception of TEE. Besides, some of them have been previously evaluated^10^.

### Statistical analysis

The statistical analysis was similar to those previously described by Prado-Nóvoa et al.^22^. T-test analyses were used to compare sample descriptive characteristics by sex. A one-way repeated-measures analysis of variance (ANOVA), with Bonferroni post-hoc tests, was used to compare measured and estimated TEE means (p < 0.05). Agreement between measured and predicted TEE was analyzed by Bland–Altman plots^30^. The association between the magnitude of the TEE and the difference between predicted and measured TEE (heteroscedasticity) was examined by regression analysis, and the slope (β) pointed when the relationship was significant (p < 0.05) in the Bland–Altman plots, for the entire sample and each sex separately. Bias was calculated as the mean of the difference between measured and predicted TEE, with Standard Deviation (SD).

Other assessments of accuracy calculated were: lower (LLOA) and upper (ULOA) limits of agreement (Formula 1 in Supplementary Material), mean absolute percent error (MAPE) (Formula 2 in Supplementary Material), mean difference as a percentage (%) (Formula 3 in Supplementary Material), root mean square error (RMSE) and its percentage (RMSE%) (Formulas 4 and 5, respectively, in Supplementary Material). In previous studies, no significant difference between means (p ≥ 0.05), a mean difference (%) ≤ 10%, and an RMSE% ≤ 10% were indicative of accuracy in predictive equations for RMR^22,31–34^. Similar references are lacking for TEE predictive equations accuracy, but the same criteria will be used in this study to describe accuracy. In addition, accuracy at an individual level was calculated as the percentage of individuals with a predicted TEE within ± 10% of the measured TEE.

One-way ANOVA analyses were used to test the effect of sex on the equation’s accuracy. The biases of the predicted TEE were examined against age, sex, BM, height, FM, FFM, percentage of FM, percentage of FFM, VM CPM, and PAL by multiple regression (General Linear Models—GLM), with backward deletion, avoiding multicollinearity. This analysis was made in the entire sample and separately by sex which allowed us to assess if our participants’ characteristics and PA were affecting the error magnitude of the estimations. Lastly, GLMs with backward deletion were also applied with measured TEE as the dependent variable in our total sample and separately by sex.

As it was expected, the PAL of our participants affected the performance of the predictive equations, so those individuals with PAL ≤ 1.89 (n = 28) were re-analyzed following the same procedures previously described. The cut point for PAL was set at 1.89 to eliminate very active subjects^35^ from the calculations. To avoid redundancy, these analyses were only repeated in the three most accurate equations in the entire sample (Plucker 3^20^, Pontzer2^3^, Vinken1^19^). These subsets of analyses may improve the applicability of our results, allowing comparisons with other populations that are more sedentary.

## Results

### Accuracy of the predictive equations in the whole sample

The characteristics of our sample are shown in Table 1. Males had a significantly higher BM, height, FFM, and %FFM, but a lower %FM compared with females (p < 0.001). RMR and TEE were also significantly higher for males (p < 0.001). However, there were no significant differences in the remainder of the characteristics (age, BMI, FM, PAEE, PAL, Steps/d, and VM CPM) between males and females.

**Table 1:** Summary characteristics of the sample.

	Total sample (n = 56)		Subjects with PAL ≤ 1.89 (n = 28)	
	♀ (n = 27)	♂ (n = 29)	♀ (n = 12)	♂ (n = 16)
Age (years)	35 ± 9	35 ± 10	33 ± 10	35 ± 11
BM (kg)	58.9 ± 7.0	73.8 ± 9.3*	60.2 ± 9.2	76.0 ± 10.4*
Height (cm)	165 ± 5	181 ± 7*	164 ± 4	183 ± 6*
BMI (kg/m^2^)	21.7 ± 1.9	22.5 ± 2.1	22.7 ± 2.5	22.6 ± 1.9
FFM (kg)	44.9 ± 4.8	60.9 ± 6.4*	43.8 ± 5.2	61.8 ± 7.0*
%FFM	76.4 ± 7.1	82.9 ± 4.9*	73.4 ± 7.9	81.7 ± 5.3*
FM (kg)	14.6 ± 5.3	13.2 ± 4.6	16.7 ± 6.9	14.6 ± 5.1
%FM	24.3 ± 6.4	17.6 ± 4.7*	27.1 ± 7.7	18.9 ± 5.1*
RMR (kcal/d)	1459 ± 144	1828 ± 1923*	1456 ± 156	1882 ± 208*
TEE (kcal/d)	2841 ± 478	3408 ± 525*	2550 ± 389	3114 ± 455*
PAEE (kcal/d)	1137 ± 329	1186 ± 516	839 ± 223^†^	920 ± 345^†^
PAL	1.97 ± 0.22 (Min. 1.53–Max. 2.48)	1.84 ± 0.34 (Min. 1.31–Max. 2.57)	1.75 ± 0.13^†^ (Min. 1.53–Max. 1.89)	1.66 ± 0.20^†^ (Min. 1.31–Max. 1.88)
Steps/d	12,781 ± 3920^#^ (Min. 4142–Max. 21,555)	12,284 ± 4108 (Min. 6064–Max. 21,712)	12,483 ± 4311.88^#^ (Min. 5106–Max. 16,662)	10,790 ± 3155 (Min. 6276–Max. 15,926)
VM CPM	806.3 ± 212.6^#^ (Min. 367.4–Max. 1157)	831.9 ± 235.2 (Min. 416.8–Max. 1198)	748.9 ± 216.3^#^ (Min. 367.4–Max. 996.1)	703.3 ± 179.6 (Min. 416.8–Max. 1029.7)

Data expressed as mean ± standard deviation.

♀: females; ♂: males; BM: body mass; BMI: Body Mass Index; FFM: fat-free mass; FM: fat mass; RMR: resting metabolic rate; TEE: total energy expenditure; PAEE: Physical activity energy expenditure; PAL: physical activity level (TEE/RMR); VM CPM: vector magnitude counts per minute per day.

*Significant differences by sex (T-Student test, p-value < 0.05).

^†^Significant difference with the total sample (T-Student test, p-value < 0.05).

^#^Three females were excluded for Steps/d and VM CPM due to accelerometry criteria for valid days.

**Figure 1:**
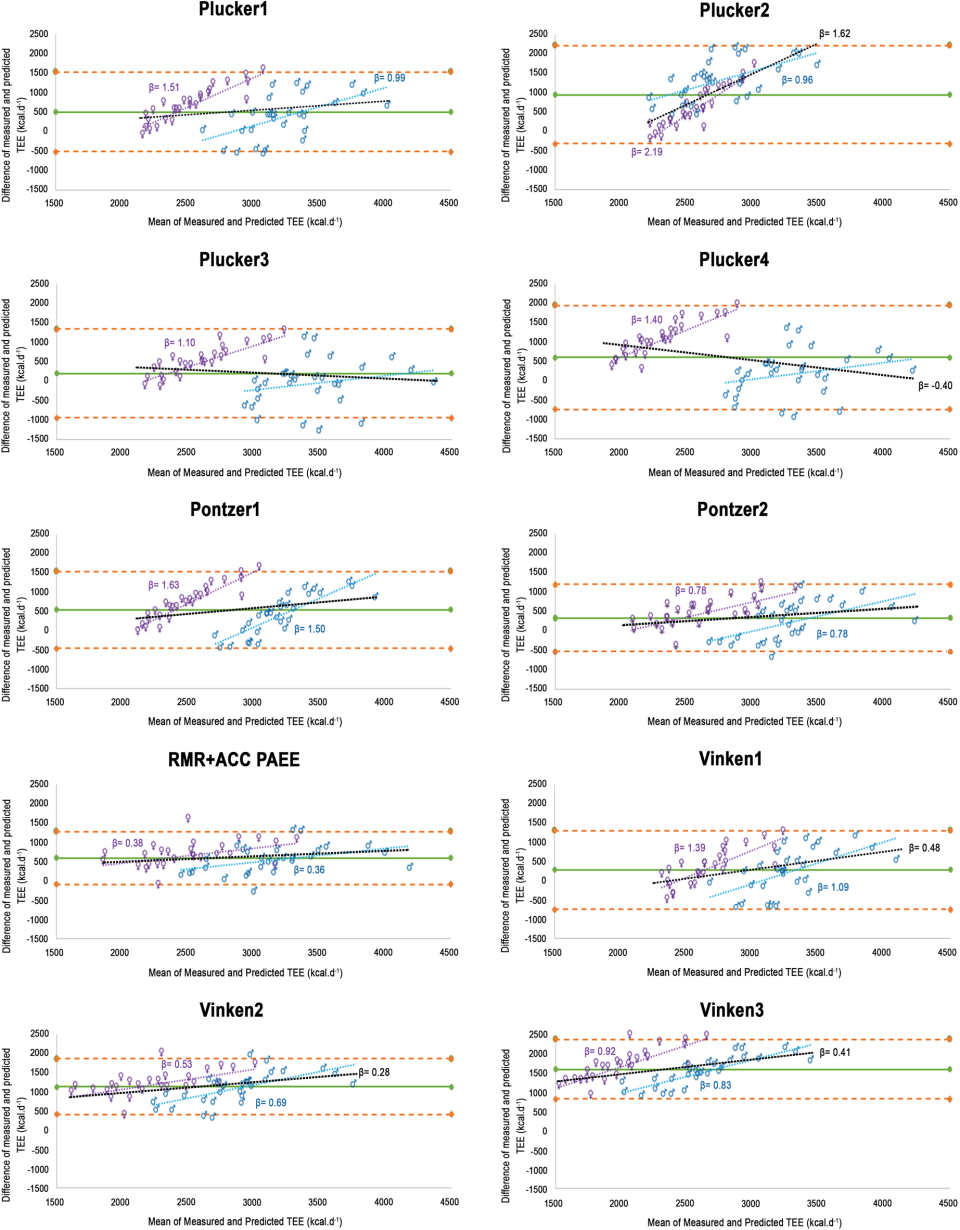
Bland–Altman plots for measured and predicted TEE applied to the whole sample (n = 56). Purple (♀ = females), blue (♂ = males), and black (both sexes) dotted lines represent the relationship between the magnitude of the TEE and the extent of error of the predictive equations by sex (homoscedasticity or heteroscedasticity). When β (slope of the line) is shown, heteroscedasticity is significant (p-value < .01). Green solid line shows the mean difference between measured and predicted TEE for each model. Orange dashed lines show the limits of agreement (Bias ± 1.96*Standard Deviation).

The comparisons between estimated and measured TEE, positive MAPE, and positive mean difference (%) indicated that all predictive models underestimated the TEE in the entire sample (Table 2) and for females (Table S2). However, the Plucker3 model was the only one overestimating the TEE of males, but with a notable individual variability (average of 68 kcal ± 613) (Fig. 1). Based on the accuracy criteria established in this study, the Plucker3 equation performed the best in the entire sample (Table 2) and better in males than females (Tables 3 and S2). Plucker4 also predicted TEE in males more accurately (Tables 3 and S2). However, all of the models applied had an RMSE% > 10%, indicating generally low performance of the equations at an individual level. Accordingly, Plucker3 showed the highest percentage of individuals with a predicted TEE within ± 10% of the measured value, close to 43% in the entire sample and 55% of the males (Tables 2 and S2). This generally indicates a lower precision of the equations.

**Table 2:** Validity and accuracy of equations to estimate TEE (kcal/day) in the whole sample and for those subjects with physical activity levels (PAL) ≤ 1.89.

		Mean ± SD	Bias (Mean ± SD)	LLOA	ULOA	MAPE	Mean difference %	RMSE	%RMSE	Accuracy (%)
Whole sample (n = 56)	Measured TEE	3134 ± 575	–	–	–	–	–	–	–	–
	Predicted TEE									
	Plucker1	2625 ± 479*	509.31 ± 524.94	− 519.57	1538.19	18.64	14.87	728.04	27.73	17.86
	Plucker2	2185 ± 229*	949.05 ± 649.40	− 323.77	2221.87	28.60	27.65	1146.68	52.48	16.07
	Plucker3	**2940 ± 646**	194.55 ± 581.19	− 944.59	1333.68	15.15	**5.16**	607.95	20.68	42.86
	Plucker4	2528 ± 788*	606.67 ± 683.69	− 733.36	1946.70	24.81	19.11	909.47	35.98	21.43
	Pontzer1	2586 ± 457*	548.30 ± 507.92	− 447.22	1543.83	18.92	16.15	744.32	28.78	19.64
	Pontzer2	2795 ± 477*	339.18 ± 442.73	− 528.58	1206.94	14.54	**9.68**	554.58	19.84	25.00
	RMR + ACC PAEE #	2548 ± 484*	628.60 ± 359.28	− 75.59	1332.80	19.87	19.33	589.49	23.14	17.86
	Vinken1	2851 ± 401*	283.63 ± 521.45	− 738.40	1305.67	15.06	**7.09**	589.49	20.68	37.50
	Vinken2	1987 ± 448*	1147.30 ± 368.48	425.07	1869.52	36.51	36.51	1204.01	60.59	0.00
	Vinken3	1522 ± 408*	1612.18 ± 397.76	832.57	2391.79	51.51	51.51	1659.67	109.05	0.00
PAL ≤ 1.89 (n = 28)	Measured TEE	2872 ± 507	–	–	–	–	–	–	–	–
	Predicted TEE									
	Plucker3	**3051 ± 969**	− 178.75 ± 502.38	− 1163.41	805.91	13.19	− **6.45**	524.71	17.20	53.57
	Pontzer2	**2828 ± 526**	44.32 ± 358.32	− 657.98	746.62	10.66	**1.03**	354.64	12.54	39.29
	Vinken1	**2931 ± 424**	− 58.37 ± 416.97	− 875.62	758.88	12.42	− **3.48**	413.59	14.11	50.00

RMR + ACC PAEE = Measured resting metabolic rate + Physical activity energy expenditure measured by accelerometry, using the standard thermic effect of food (0.9). Data expressed as mean ± standard deviation (SD). Bias = mean of the difference between measured and predicted TEE, positive values indicate underestimation, negative values indicate overestimation; LLOA = lower limit of agreement; ULOA = upper limit of agreement; MAPE = mean absolute percent error; Mean difference % = percentage of the difference between measured and predicted TEE; RMSE = root mean square of error; %RMSE = Percentage of root mean square of error; Accuracy (%) = percentage of subjects with a predicted TEE within ± 10% of the measured value. *Significant difference between predicted and measured TEE (Bonferroni post hoc test, p-value < 0.05). Values in bold represent accomplished criteria to consider an equation accurate: no significant difference between measured and predicted TEE; and mean difference % ≤ 10%. #Three females were excluded due to accelerometry criteria for valid days.

**Table 3:** Summary of equations meeting 2 criteria to be considered accurate when applied to the whole sample and dichotomized by sex (♀ = Females; ♂ = Males) and also for those subjects with physical activity levels (PAL) ≤ 1.89.

		PLUCKER1	PLUCKER2	PLUCKER3	PLUCKER4	PONTZER1	PONTZER2	RMR + ACC PAEE	VINKEN1	VINKEN2	VINKEN3
Total sample	♀ and ♂ (n = 56)			✓							
	♀ (n = 27)										
	♂ (n = 29)			✓	✓						
Subjects with PAL ≤ 1.89	♀&♂ (n = 28)			✓			✓		✓		
	♀ (n = 12)			✓					✓		
	♂ (n = 16)						✓		✓		

In this subset of subjects with PALs ≤ 1.89, only Plucker3, Pontzer2, and Vinken1 models were tested (dark gray headings). Criteria accomplished: no significant difference between measured and predicted TEE, mean difference (%) ≤ 10%.

All the equations showed large limits of agreement and RMSE (Table 2 and Fig. 1). Some equations showed heteroscedasticity (p < 0.01, black dotted line in Fig. 1), especially when each sex was considered separately (purple [females] and blue [males] dotted lines in Fig. 1). Sex influenced some indicators of accuracy; the performance of the equations was generally poorer when applied to females: significantly higher bias, MAPE, mean difference %, and lower accuracy (%) (see details in Table S2).

GLM regressions showed that in addition to age or body composition, physical activity (VM CPM and/or PAL) impacted the bias of the models in the whole sample and by sex (Table S3). Therefore, the error of the estimations was generally higher for more active participants.

On the other hand, GLM with measured TEE as the dependent variable showed BM, FM, and VM CPM predicted 61% of the variability in our total sample (Table S4). For males, 73% of TEE variability was explained by FFM and VM CPM. However, only 52% of TEE variability was explained for females, with RMR as the only significant variable in the model (Table S4).

### Accuracy of the predictive equations in subjects with PAL ≤ 1.89

When the subset of subjects with PAL ≤ 1.89 were compared to the whole sample, significant differences were only found for PAEE and the average PAL (significantly lower, p < 0.05) (Table 1). The statistical differences detected by sex in the whole sample remained in this subset of participants (Table 1).

The three equations applied (Plucker3, Pontzer2, and Vinken1) performed significantly better in these subjects than when applied to the entire sample (Table 2). Although Pontzer2 was the only equation slightly underestimating TEE (average of − 44 kcal ± 358), predicted TEE averages were not significantly different from measured TEE in any equation. The mean difference (%) was < 10% for the three equations. The only accuracy metric not accomplished was %RMSE ≤ 10%, although the results were better than for the entire sample. Pontzer2, followed by Vinken1, were the most accurate equations (lower bias, mean difference%, and %RMSE, Table 2).

**Figure 2:**
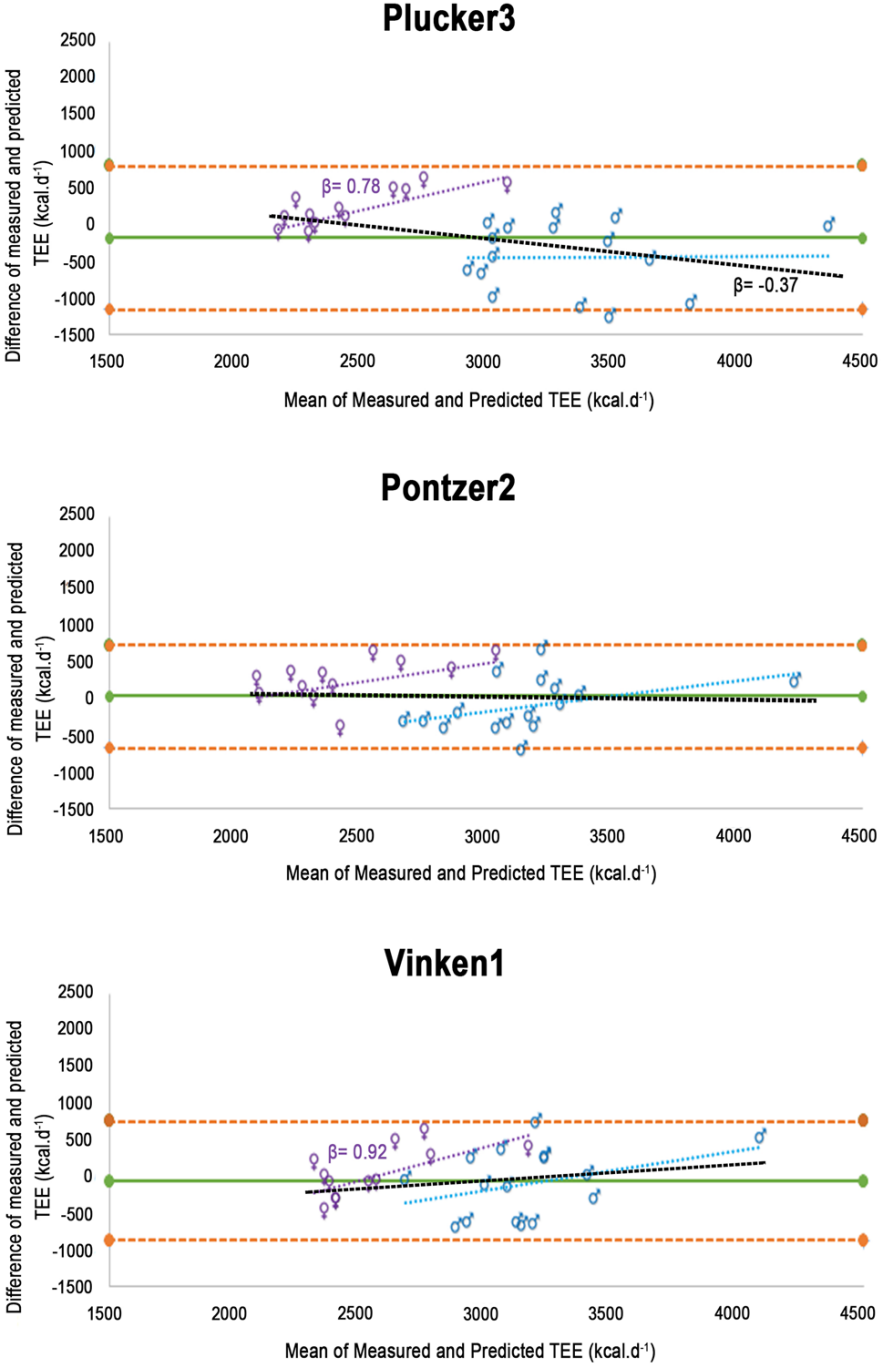
Bland–Altman plots for measured and predicted TEE for those subjects with physical activity levels ≤ 1.89 (n = 28). The three equations shown are the ones with a better performance in the entire sample. Purple (♀ = females), blue (♂ = males), and black (both sexes) dotted lines represent the relationship between the magnitude of the TEE and the extent of error of the predictive equations by sex (homoscedasticity or heteroscedasticity). When β (slope of the line) is shown, heteroscedasticity is significant (p-value < .01). Green solid line shows the mean difference between measured and predicted TEE for each model. Orange dashed lines show the limits of agreement (Bias ± 1.96*Standard Deviation).

Lower performance at the individual level was still detected in these subjects (large limits of agreement) (Table 2), but Pontzer2 did not show heteroscedasticity when applied to these subjects, and heteroscedasticity was reduced for Vinken1 (Fig. 2). The percentage of individuals with a predicted TEE within ± 10% of the measured value (precision) was close to 50% for both sexes, and notably higher (67%) for females with the Plucker3 equation (Accuracy (%), Tables 2 and S2). Although higher in this subset, the precision of the equations can still be considered low.

Sex influenced some indicators of accuracy (Table S2), so Plucker3 and Vinken1 were more accurate for females and Pontzer3 for males (Tables S2 and 3). In this subset of analyses, Vinken1 was the only equation being accurate for both sexes (Tables S2 and 3).

GLM regressions for the bias of the models also showed that, besides characteristics like age, BM, or body composition, physical activity (VM CPM and/or PAL) positively influenced the error of the estimations (Table S3). On the other hand, measured TEE was more predictable in this subset of individuals (GLM), with AdjR^2^ ranging from 73.59 to 76.98%, using BM, RMR, and VM CPM as independent variables (see details in Table S4).

## Discussion

The major finding of this study was that all of the models applied on average underestimated the TEE in our entire sample, and none met all the criteria to be considered accurate. This underestimation was usually greater with increasing TEE, which in our sample also indicated higher physical activity levels (as PAL or as VM CPM). The equation published by Plucker et al.^20^ based on age, body mass, height, and RMR was the most accurate in predicting the TEE in our entire sample (average bias of 195 kcal). However, the accuracy and precision of the equations applied were significantly improved when less active individuals (PAL ≤ 1.89) were considered separately. In this case, Pontzer2^3^ (underestimating an average of 44 kcal) and Vinken1^19^ (overestimating an average of 58 kcal) were the most accurate. The latter observation was independent of the sex of the participants.

The RMSE% > 10% reflected the sizable errors at the individual level for all the equations. The heteroscedasticity detected (Figs. 1 and 2) and the influence of our participant's PA on the equation's accuracy (Table S3) contributed to the differences between our population and those used to develop the predictive models (Table S1). Our participants were younger with lower BMI and notably higher TEE than those in Plucker et al.^20^, Pontzer et al.^3^, and Vinken et al.^19^ (Tables 1 and S1). The latter was a consequence of the higher levels of PA in our sample.

It is noteworthy to highlight the poor performance of the equations that included accelerometry-derived PAEE (Vinken2 and Vinken3, Table 2 and Fig. 1). The limitations of considering accelerometry PAEE to predict TEE include the use of models from different manufacturers, different wear locations, and/or sampling frequency (see references in Fernández-Verdejo and Galgani^10^). Therefore, the lack of standardization may reduce the applicability of these predictive equations. In this regard, our findings (RMR + ACC PAEE in Table 2) are consistent with a significant underestimation of TEE derived from accelerometry PAEE in free-living conditions^11,36^. As previously mentioned^10^, improving TEE estimations by including objectively measured PA remains a challenge, and accelerometry-derived PAEE accuracy is highly variable^13^. Standardization in the units of measurement, sharing the technical specifications and computational methods of the manufacturers, and better calibrations of PAEE against gold-standard techniques may improve the performance of these equations in future studies.

Interestingly, the three metrics of accuracy were differentially impacted by sex in the equations evaluated (Table S2). Contrary to the RMR prediction in our sample^22^, the equations evaluated in the present study were more accurate in males than females (Table S2), especially when the whole sample is considered (Table S4). Finally, subject characteristics and PA accounted for a greater portion of the variability in bias for males than females (Table S3). Future studies need to address sex in the development of equations to predict TEE, as has been suggested for RMR prediction^22,37^.

As also pointed out by Fernández-Verdejo and Gaglani^10^ and Macena et al.^38^, there is a gap in the literature validating TEE equations’ accuracy across different populations. We are aware that some of the equations with higher accuracy and precision in our study may not easily apply for others due to the inclusion of the RMR or body composition parameters. However, in our sample, these are also the factors remaining in the models to predict TEE (Table S4). This agrees with previous studies^20,39–41^ (but see Tudor-Locke et al.^42^), but challenges the possibility of accurately predicting TEE based on simple factors such as age, sex, height, and body mass, especially if individuals with different physical activity levels are considered (Table 2).

Although finding a unique predictive model for energy requirements across populations may be difficult^20^ and predictive equations perform poorly at an individual level (low precision), these equations are still essential for many clinical interventions^20,43^, i.e. to determine nutritional needs, target energy balance, or health improvements and lifestyle changes. Moreover, the average bias of the equations performing best in our sample was less than 195 kcal (Table 2). Together with other metrics of accuracy in Table 2, this indicates that these equations may be superior to other techniques to approximate TEE, like self-reported energy intake^44^, motion sensors^45^, and heart rate monitoring^46^, without the necessity of individual calibrations.

### Practical applications and strengths

Our analyses indicated that when a large variability in physical activity levels (from sedentary to very active) was considered, Plucker3 equation was most accurate in predicting TEE at a population level, but less so at an individual level (low precision) and for females alone. Additionally, RMR is included in Plucker3 model, which may not be available or, if estimated, may add error to TEE estimation.

The Pontzer2 equation was the most accurate model, particularly for males, when less active individuals (PAL ≤ 1.89) were considered separately. However, the Vinken1 equation was the most accurate, for both males and females, among the equations that did not rely on body composition. Nonetheless, caution must be taken as our analyses suggested that the error of the predictions was influenced by sample parameters like body composition and physical activity.

There are several strengths of our study. First, we included gold-standard techniques for the measurement of body composition and both RMR and TEE. In addition, we quantified habitual physical activity of our participants over a 14-day period using accelerometry^42,47,48^. Second, the inclusion of similar numbers of males and females and a uniform distribution of physical activity levels allowed us to consider if these variables impact predictions. Third, body mass and composition stability were documented over the measurement period, thus avoiding a potential confound of energy imbalance. Lastly, we utilized objective metrics for evaluating the accuracy of published prediction equations and, in so doing, may serve as a reference to others seeking to develop and validate new equations.

### Limitations of the study

There are some limitations that should be considered. First, our sample size was relatively small. As such, different results may be obtained in a larger sample. Second, the participants in our study were primarily Caucasian, young, with normal weight, and generally more active than the US population^49^. As such, the accuracy of the predictive equations may be different when applied to other groups. Finally, the assumed respiratory quotients (RQ) used to calculate TEE in the DLW calculations^25^ may have impacted the accuracy of the predictive equations.

## Conclusions

The present study demonstrated that available published equations tended to underestimate TEE in our sample. Although some models were accurate in predicting TEE across a wide spectrum of habitual physical activity and in the less active participants (i.e., PAL ≤ 1.89), precise prediction of TEE at an individual level remains a challenge. More studies are needed to develop and validate predictive equations that do not rely on a classic additive conception of the TEE. The validation of these equations in diverse populations is necessary to improve application.

### Supplementary Information

Supplementary Information.

## Data Availability

The datasets generated during the current study are available from the corresponding author on reasonable request.
