# Supplementary material for: Validity of predictive equations for total energy expenditure against doubly labeled water
Source: Sci Rep. 2024 Jul 8;14:15754. doi: 10.1038/s41598-024-66767-7 (PMC11231257; doi:10.1038/s41598-024-66767-7)
Supplement: Supplementary file 1 — Supplementary Information. [file 41598_2024_66767_MOESM1_ESM.docx]

**Supplementary information**

Formulas used to evaluate predictive equations accuracy:

1. Lower limits of agreement ($LLOA)=Bias-\left( 1.96*SD \right)$

Upper limits of agreement ($ULOA)=Bias+\left( 1.96*SD \right)$

2. Mean absolute precent error (MAPE)$=\left( \frac{1}{n} \right)\sum_{t=1}^{n} \left( \frac{Measured TEE-Predicted TEE}{Measured TEE} \right)$

3. Mean difference (%)$=\left( \frac{Measured TEE-Predicted TEE}{Measured TEE} \right)*100$

4. Root mean square of error (RMSE)$=\sqrt{\frac{\sum_{i=1}^{n} {(Measured TEE-Predicted TEE)}^{2}}{n}}$

5. Percentage of root mean square of error (RMSE%) $=\left( \frac{RMSE}{Predicted TEE} \right)*100$

| Equation applied | Model ID | Sex | Age (years) | Model | | | Population | Methodology |
| --- | --- | --- | --- | --- | --- | --- | --- | --- |
| Plucker et al. (2018) [Model #1] | Plucker1 | ♀ | Adults | TEE | = 563.78 − 8.79 * Age + 14.31 * BM + 6.58 * Height | Adj R^2^= 0.68  RMSE= 244.58 | n=119  Data from Pennington Biomedical Research Center database.  ♀ BMI= 26.86 ±4.79  Age= 53.96 ±24.79  TEE= 2,174.49 ±442.87  ♂ BMI= 27.70 ±3.11  Age= 58.55 ±24.43  TEE= 2,946.61 ±793.20 | TEE measured by DLW and a metabolic chamber  Body composition: DXA |
|  |  | ♂ |  |  | = 3916.32 − 19.21 * Age + 27.18 * BM − 12.29 * Height | Adj R^2^= 0.65  RMSE= 453.80 |  |  |
| Plucker et al. (2018)  [Model #2] | Plucker2 | ♀ |  |  | = 1293.24 − 6.73 * Age − 18.10 * BM − 2.89 * Height + 48.26 * FM + 27.03 * FFM | Adj R^2^= 0.72  RMSE= 227.39 |  |  |
|  |  | ♂ |  |  | = 5791.79 − 16.318 * Age + 22.60 * BM − 31.90 * Height + 6.14 * FM + 14.57 * FFM | Adj R^2^= 0.66  RMSE= 437.10 |  |  |
| Plucker et al (2018)  [Model #3] | Plucker3 | ♀ |  |  | = 179.17 − 5.99 * Age + 6.14 * BM + 4.80 * Height + 0.85 * RMR | Adj R2= 0.73  RMSE= 223.43 |  |  |
|  |  | ♂ |  |  | = 2764.38 − 11.53 * Age + 6.78 * BM − 16.73 * Height + 1.99 * RMR | Adj R^2^= 0.73  RMSE= 401.83 |  |  |
| Plucker et al (2018)  [Model #4] | Plucker4 | ♀ |  |  | = 715.86 − 5.18 * Age − 17.47 * BM − 0.63 * Height + 38.15 * FM + 19.56 *FFM + 0.65 * RMR | Adj R^2^= 0.74  RMSE= 215.54 |  |  |
|  |  | ♂ |  |  | = 3497.2283 − 11.3305 * Age + 10.2868 * BM − 23.0481 * Height − 4.1253 * FM + 2.9667 * FFM + 1.8602 * RMR | Adj R^2^= 0.72  RMSE= 399.73 |  |  |
| Pontzer et al. (2021)  [Model #1] | Pontzer1 | ♀&♂ | 20-60 | TEE  (MJ) | = 5.984 + 0.065 * BM + 2.669 * Sex (females 0; males 1) – 0.025*Age | Adj R^2^= 0.482  SEE= 2.032 | n= 2,805  Pooled values:  Age= 37.9 ±10.4  BM= 76.0 ±18.6  BMI= 26.6 ±5.9  TEE= 2,642 ±675 | DLW |
| Pontzer et al. (2021)  [Model #2] | Pontzer2 |  | 20-60 |  | = - 1.102 + 0.916 * ln (FFM) – 0.03 * ln (FM) | Adj R^2^= 0.65  SEE= 0.142 |  |  |
| RMR + ACC PAEE | RMR + ACC PAEE | ♀&♂ | 20-58 | TEE | = RMR + ACC PAEE + TEF | - | This study | Calorimetry for RMR  Tri-axial accelerometry for PAEE  Standard thermic effect of food (10%) |
| Vinken et al. (1999)  [Model #1] | Vinken1 | ♀&♂ | 18-81 | TEE  (MJ) | = 7.377 - 0.073 * Age + 0.0806 * BM + 0.0135 * Height – 1.363 * Sex (females 1; males 0) | Adj R2= 0.64  SEE= 1.80 | n=93  Pooled values:  Age= 50.2 ±21.0  BM= 68.1 ±12.6  BMI= 23.8 ±3.3  TEE= 2,567 ±697 | DLW  Activity Monitors (accelerometers)  Body composition: underwater weighing with correction for measured residual lung volume. |
| Vinken et al. (1999)  [Model #2] | Vinken2 |  |  | TEE  (MJ) | = 0.107 + 0.612 * activity monitor - 0.0577 * %FM + 1.149 * RMR | Adj R2= 0.78  SEE= 1.55 |  |  |
| Vinken et al. (1999)  [Model #3] | Vinken3 |  |  | TEE  (MJ) | = 5.223 - 0.033 * Age + 0.040 * BM  - 0.0093 * Height - 1.684 * Sex (females 1; males 0) + 0.76 * activity monitor | Adj R2= 0.76  SEE= 1.65 |  |  |

Table S1. Characteristics of the different equations applied to estimate total energy expenditure (TEE), in kilocalories/24h except when indicated in other units. Model ID: identification given to the models applied in this study. RMR+ ACC PAEE= Measured resting metabolic rate + Physical activity energy expenditure estimated by accelerometry + the standard thermic effect of food (10%). n= sample size; ♀= Females; ♂= Males. BM= body mass in kilograms; Height in centimeters; Age in ears; FFM= fat-free mass in kilograms; FM= fat mass in kilograms. RMR= Resting Metabolic Rate. BMI= Body mass index (kg/m^2^). DLW= Doubly Labelled Water technique. DXA= dual energy X-ray absorptiometry.

|  |  | Mean (kcal/day) ± SD | | Bias (Mean ± SD) | | | MAPE | | | Mean difference % | | | RMSE | | %RMSE | | Accuracy (%) | |
| --- | --- | --- | --- | --- | --- | --- | --- | --- | --- | --- | --- | --- | --- | --- | --- | --- | --- | --- |
|  |  | ♀ (n=27) | ♂ (n=29) | ♀ | ♂ | Sex effect (F-ratio) | ♀ | ♂ | Sex effect (F-ratio) | ♀ | ♂ | Sex effect (F-ratio) | ♀ | ♂ | ♀ | ♂ | ♀ | ♂ |
| Whole sample (n=56) | Measured TEE | 2,841 ±478.44 | 3,408 ±525.05 | - | - | - | - | - | - | - | - | - | - | - | - | - | - | - |
|  | Predicted TEE | | | | | | | | | | | | | | | | | |
|  | Plucker1 | 2,189 ±117.92* | 3,033 ±283.47* | 653.87 ±448.12 | 374.72 ±562.06 | 4.18 | 21.69 ±11.56 | 15.79 ±8.99 | 4.59 | 21.14 ±12.57 | **9.04 ±15.95** | 9.85† | 787.98 | 667.41 | 36.00 | 22.00 | 18.52 | 20.69 |
|  | Plucker2 | 2,283 ±103.30* | 2,094 ±273.77* | 557.42  ±528.97 | 1,313.67 ±531.94 | 28.41† | 19.18 ±12.79 | 37.37 ±11.56 | 32.03† | 17.21 ±15.42 | 37.37 ±11.26 | 31.52† | 767.68 | 1,413.84 | 33.63 | 67.52 | 33.33 | 0.00 |
|  | Plucker3 | 2,364 ±169.66* | **3,476 ±419.68** | 476.71 ±388.22 | -68.16 ±612.62 | 15.54† | 16.41 ±9.45 | 13.97 ±13.50 | 0.60 | 15.13 ±11.46 | **-4.13**  **±19.15** | 20.45† | 610.24 | 605.81 | 25.81 | 17.43 | 25.93 | 55.17 |
|  | Plucker4 | 1,782 ±142.00* | **3,222 ±405.22** | 1,058.76 ±448.06 | 185.76 ±592.60 | 38.23† | 35.82 ±10.36 | 14.55 ±10.07 | 60.64† | 35.82 ±10.36 | **3.56**  **±17.54** | 68.91† | 1146.43 | 611.20 | 64.33 | 18.97 | 0.00 | 37.93 |
|  | Pontzer1 | 2,138 ±97.38* | 3,004 ±159.12* | 702.96 ±456.02 | 404.32 ±518.77 | 5.20† | 23.03 ±12.08 | 15.09 ±8.36 | 8.28† | 22.86 ±11.41 | **9.90 ±14.29** | 13.04† | 833.31 | 650.63 | 38.98 | 21.66 | 14.81 | 24.13 |
|  | Pontzer2 | 2,389 ±239.61* | 3,174 ±293.61* | 451.84 ±363.38 | 234.29 ±488.65 | 3.53 | 16.08 ±8.52 | 13.11 ±6.95 | 2.05 | 14.53 ±11.04 | **5.17 ±14.10** | 7.58† | 575.60 | 534.26 | 24.09 | 16.83 | 18.52 | 27.58 |
|  | RMR + ACC PAEE | 2,191 ±334.04*# | 2,843 ±380.17* | 706.11 ±346.50 | 564.46 ±362.90 | 2.08 | 24.08 ±8.68 | 16.40 ±8.11 | 11.05† | 23.60 ±9.95 | 15.79 ±9.27 | 8.72† | 783.36 | 667.66 | 35.75 | 23.48 | 4.17 | 31.03 |
|  | Vinken1 | 2,509 ±163.76* | 3,169 ±270.12* | 332.16 ±469.25 | 238.45 ±570.31 | 0.45 | 15.33 ±9.07 | 14.81 ±8.97 | 0.05 | **9.47 ±15.28** | **4.87 ±16.83** | 1.14 | 567.78 | 609.01 | 22.63 | 19.22 | 33.33 | 37.93 |
|  | Vinken2 | 1,637 ±296.76* | 2,313 ±290.78* | 1,203.97 ±320.60 | 1,094.53 ±406.55 | 1.24 | 42.09 ±7.60 | 31.32 ±8.55 | 24.68† | 42.09 ±7.60 | 31.32 ±8.55 | 24.68† | 1,244.40 | 1,165.15 | 70.02 | 50.37 | 0.00 | 0.00 |
|  | Vinken3 | 1,159 ±179.83* | 1,861 ±226.17* | 1,682.21 ±412.00 | 1,546.97 ±379051 | 1.64 | 58.63 ±6.48 | 44.87 ±5.66 | 71.97† | 58.63 ±6.48 | 44.87 ±5.66 | 71.97† | 1,730.11 | 1,591.28 | 136.89 | 85.51 | 0.00 | 0.00 |
| PAL ≤1.89 (n=28) | Measured TEE | ♀ (n=12)  2,550 ±388.79 | ♂ (n=16)  3,114 ±454.91 | - | - | - | - | - | - | - | - | - | - | - | - | - | - | - |
|  | Predicted TEE | | | | | | | | | | | | | | | | | |
|  | Plucker3 | **2,373 ±183.01** | 3,560 ±451.23* | 177.12 ±260.58 | -445.65 ±476.66 | 16.64† | 8.78 ±6.29 | 16.54 ±15.92 | 2.58 | **5.83 ±9.24** | -15.66  ±16.85 | 15.84† | 305.96 | 604.56 | 12.89 | 16.98 | 66.67 | 50.00 |
|  | Pontzer2 | 2,329 ±252.30* | **3,202 ±320.43** | 220.87 ±289.54 | -88.09 ±354.95 | 6.05† | 11.16 ±6.88 | 10.28 ±5.94 | 0.13 | **7.68 ±10.91** | **-3.95 ±11.46** | 7.34† | 354.44 | 354.79 | 15.22 | 11.08 | 33.33 | 43.75 |
|  | Vinken1 | **2,554 ±187.79** | **3,213 ±315.33** | -3.72 ±346.22 | -99.36 ±469.96 | 0.35 | 11.44 ±6.86 | 13.15 ±9.06 | 0.30 | **-1.76 ±13.65** | **-4.77 ±15.57** | 0.28 | 311.50 | 465.76 | 12.20 | 14.50 | 50.00 | 50.00 |

Table S2. Accuracy of TEE predictive models applied to the sample divided by sex (whole and subjects with physical activity levels (PAL) ≤1.89) and results of One-Way ANOVA analysis on the effect of sex on equations bias, MAPE, and Mean difference % (F-ratio). ♀= Females; ♂= Males. Bias= mean of the difference between measured and predicted TEE, positive values indicate underestimation, negative values indicate overestimation; MAPE= mean absolute percent error; Mean difference %= percentage of the difference between measured and predicted TEE; RMSE= root mean square of error; %RMSE= Percentage of root mean square of error; Accuracy (%)= percentage of subjects whit a predicted TEE within ±10% of the measured value. *Significant difference between predicted and measured TEE (Bonferroni *post hoc* test, p-value <0.05). †Significant effect of sex on the variable analyzed (One Way ANOVA, p-value <0.05). Values in bold represent accomplished criteria for an accurate predictive equation: no significant difference between measured and predicted TEE, mean difference (%) ≤10%. #Three females were excluded due to accelerometry criteria for valid days.

|  | | ♀&♂ | | | | | | ♀ | | | | | | | | | ♂ | | | | | |
| --- | --- | --- | --- | --- | --- | --- | --- | --- | --- | --- | --- | --- | --- | --- | --- | --- | --- | --- | --- | --- | --- | --- |
| Equations ‘bias (Whole sample) | Plucker1 | **Factors** | **β** | **Std. err.** | **f** | **p** | **V.I.F.** | **Factors** | **β** | **Std. err.** | | **f** | **p** | | **V.I.F.** | | **Factors** | **β** | **Std. err.** | **f** | **p** | **V.I.F.** |
|  |  | **Intercept** | -3377.00 | 333.05 | 63.73 | <0.001 |  | **Intercept** | -3157.37 | 483.41 | | 40.45 | <0.001 | |  |  | **Intercept** | -2657.75 | 259.45 | 59.68 | <0.001 |  |
|  |  | **Sex** | 214.18 | 47.74 | 20.13 | <0.001 | 2.96 | **BM** | 21.25 | 5.99 | | 12.57 | 0.002 | | 1.34 | | **Age** | 13.42 | 4.11 | 10.66 | 0.003 | 1.11 |
|  |  | **Age** | 9.23 | 2.99 | 9.57 | 0.002 | 1.04 | **%FM** | -17.54 | 7.41 | | 5.60 | 0.028 | | 1.57 | | **VM CPM** | 0.73 | 0.25 | 8.20 | 0.008 | 2.32 |
|  |  | **FFM** | 12.96 | 5.00 | 6.71 | 0.013 | 3.00 | **PAL** | 1539.77 | 174.46 | | 77.90 | <0.001 | | 1.20 | | **PAL** | 1041.91 | 187.79 | 30.78 | <0.001 | 2.25 |
|  |  | **VM CPM** | 0.47 | 0.15 | 9.55 | 0.003 | 1.52 | ***model*** | **N** | **SEE** | | **df** | **AdjR^2^** | |  | | ***model*** | **N** | **SEE** | **df** | **AdjR^2^** |  |
|  |  | **PAL** | 1309.16 | 122.91 | 113.45 | <0.001 | 1.53 |  | 24 | 176.63 | | 23 | 83.73 | |  |  |  | 29 | 208.21 | 28 | 86.28 |  |
|  |  | ***model*** | **N** | **SEE** | **df** | **AdjR^2^** |  |  | | | | | | | |  |  | | | | | |
|  |  |  | 53 | 200.98 | 52 | 85.78 |  |  |  |  |  |  |  |  |  |  |  |  |  |  |  |  |
|  | Plucker2 | **Factors** | **β** | **Std. err.** | **f** | **p** | **V.I.F.** | **Factors** | **β** | **Std. err.** | | **f** | **p** | | **V.I.F.** | | **Factors** | **β** | **Std. err.** | **f** | **p** | **V.I.F.** |
|  |  | **Intercept** | -7167.94 | 1001.9 | 63.81 | <0.001 |  | **Intercept** | -5196.35 | 628.69 | | 31.22 | <0.001 | |  |  | **Intercept** | -6638.29 | 1139.64 | 40.91 | <0.001 |  |
|  |  | **Sex** | -174.37 | 55.14 | 10.00 | 0.003 | 2.86 | **FM** | 22.32 | 9.77 | | 5.22 | 0.033 | | 1.14 | | **Age** | 10.20 | 3.69 | 7.66 | 0.011 | 1.12 |
|  |  | **Age** | 9.11 | 3.51 | 6.74 | 0.013 | 1.04 | **FFM** | 51.11 | 10.76 | | 22.55 | <0.001 | | 1.07 | | **Height** | 29.43 | 6.27 | 22.02 | <0.001 | 1.43 |
|  |  | **Height** | 28.35 | 5.49 | 26.64 | <0.001 | 2.81 | **PAL** | 1615.42 | 234.06 | | 47.63 | <0.001 | | 1.22 | | **FM** | -18.54 | 8.92 | 4.32 | 0.049 | 1.37 |
|  |  | **VM CPM** | 0.56 | 0.18 | 9.71 | 0.003 | 1.50 | ***model*** | **N** | **SEE** | | **df** | **AdjR^2^** | |  | | **VM CPM** | 0.87 | 0.24 | 13.10 | 0.001 | 2.56 |
|  |  | **PAL** | 1278.50 | 144.33 | 78.47 | <0.001 | 1.53 |  | 24 | 234.59 | | 23 | 79.77 | |  |  | **PAL** | 958.12 | 174.46 | 30.16 | <0.001 | 2.42 |
|  |  | ***model*** | **N** | **SEE** | **df** | **AdjR^2^** |  |  | | |  | | |  | |  | ***model*** | **N** | **SEE** | **df** | **AdjR^2^** |  |
|  |  |  | 53 | 236.34 | 52 | 85.79 |  |  |  |  |  | | |  |  |  |  | 29 | 186.61 | 28 | 87.69 |  |
|  | Plucker3 | **Factors** | **β** | **Std. err.** | **f** | **p** | **V.I.F.** | **Factors** | **β** | **Std. err.** | | **f** | **p** | | **V.I.F.** | | **Factors** | **β** | **Std. err.** | **f** | **p** | **V.I.F.** |
|  |  | **Intercept** | -4514.30 | 499.20 | 257.74 | <0.001 |  | **Intercept** | -3144.01 | 262.18 | | 130.66 | <0.001 | |  |  | **Intercept** | -5802.35 | 457.02 | 507.21 | <0.001 |  |
|  |  | **Sex** | 272.22 | 27.47 | 98.17 | <0.001 | 2.86 | **FFM** | 17.43 | 5.01 | | 12.13 | 0.002 | | 1.07 | | **Age** | 12.66 | 1.39 | 82.90 | <0.001 | 1.08 |
|  |  | **Age** | 8.53 | 1.75 | 23.79 | <0.001 | 1.04 | **PAL** | 1463.68 | 102.11 | | 205.49 | <0.001 | | 1.07 | | **BM** | -6.37 | 2.00 | 10.15 | 0.004 | 1.91 |
|  |  | **Height** | 6.32 | 2.74 | 5.33 | 0.025 | 2.81 | ***model*** | **N** | **SEE** | | **df** | **AdjR^2^** | |  | | **Height** | 12.96 | 2.84 | 20.89 | <0.001 | 1.99 |
|  |  | **VM CPM** | 0.19 | 0.09 | 4.64 | 0.036 | 1.50 |  | 24 | 109.21 | | 23 | 91.85 | |  |  | **PAL** | 1821.27 | 46.27 | 1549.6 | <0.001 | 1.16 |
|  |  | **PAL** | 1660.55 | 71.91 | 533.23 | <0.001 | 1.53 |  | | | | | | |  |  | ***model*** | **N** | **SEE** | **df** | **AdjR^2^** |  |
|  |  | ***model*** | **N** | **SEE** | **df** | **AdjR^2^** |  |  | | | | | | | | |  | 29 | 71.55 | 28 | 98.64 |  |
|  |  |  | 53 | 117.76 | 52 | 96.11 |  |  |  |  |  |  |  |  |  |  |  | | | | |  |
|  | Plucker4 | **Factors** | **β** | **Std. err.** | **f** | **p** | **V.I.F.** | **Factors** | **β** | **Std. err.** | | **f** | **p** | | **V.I.F.** | | **Factors** | **β** | **Std. err.** | **f** | **p** | **V.I.F.** |
|  |  | **Intercept** | -5349.76 | 570.38 | 238.19 | <0.001 |  | **Intercept** | -3293.33 | 340.83 | | 101.55 | <0.001 | |  |  | **Intercept** | -6142.94 | 445.47 | 486.55 | <0.001 |  |
|  |  | **Sex** | 520.98 | 33.20 | 246.30 | <0.001 | 3.32 | **FFM** | 28.19+ | 6.51 | | 18.76 | <0.001 | | 1.07 | | **Age** | 12.10 | 1.37 | 77.69 | <0.001 | 1.08 |
|  |  | **Age** | 7.51 | 2.03 | 13.68 | 0.006 | 1.12 | **PAL** | 1590.36 | 132.74 | | 143.55 | <0.001 | | 1.07 | | **Height** | 17.43 | 2.79 | 13.66 | <0.001 | 1.98 |
|  |  | **Height** | 15.18 | 3.28 | 21.38 | <0.001 | 3.23 | ***model*** | **N** | **SEE** | | **df** | **AdjR^2^** | |  | | **FFM** | -10.42 | 2.82 | 13.66 | 0.001 | 1.83 |
|  |  | **FM** | -12.14 | 4.41 | 7.59 | 0.008 | 1.42 |  | 24 | 141.97 | | 23 | 89.74 | |  |  | **PAL** | 1806.62 | 46.23 | 1527.02 | <0.001 | 1.18 |
|  |  | **VM CPM** | 0.25 | 0.10 | 6.15 | 0.017 | 1.53 |  | | |  | | |  | |  | ***model*** | **N** | **SEE** | **df** | **AdjR^2^** |  |
|  |  | **PAL** | 1598.03 | 83.04 | 370.37 | <0.001 | 1.62 |  |  |  |  | | |  |  |  |  | 29 | 70.65 | 28 | 98.56 |  |
|  |  | ***model*** | **N** | **SEE** | **df** | **AdjR^2^** |  |  | | | | | | | | |  |  | |  | | |
|  |  |  | 53 | 131.97 | 52 | 96.48 |  |  |  |  |  |  |  |  |  |  |  |  |  |  |  |  |
|  | Pontzer1 | **Factors** | **β** | **Std. err.** | **f** | **p** | **V.I.F.** | **Factors** | **β** | **Std. err.** | | **f** | **p** | | **V.I.F.** | | **Factors** | **β** | **Std. err.** | **f** | **p** | **V.I.F.** |
|  |  | **Intercept** | -3294.41 | 307.61 | 83.92 | <0.001 |  | **Intercept** | -3646.82 | 432.97 | | 61.23 | <0.001 | |  |  | **Intercept** | -3233.18 | 395.93 | 69.33 | <0.001 |  |
|  |  | **Sex** | 270.29 | 45.15 | 35.83 | <0.001 | 2.96 | **FFM** | 30.48 | 8.27 | | 13.59 | 0.001 | | 1.07 | | **FFM** | 17.27 | 5.32 | 10.54 | 0.003 | 1.00 |
|  |  | **FFM** | 18.56 | 4.73 | 15.39 | <0.001 | 3.00 | **PAL** | 1536.86 | 168.62 | | 83.07 | <0.001 | | 1.07 | | **VM CPM** | 0.89 | 0.22 | 16.87 | <0.001 | 2.25 |
|  |  | **VM CPM** | 0.47 | 0.15 | 10.45 | 0.002 | 1.51 | ***model*** | ***N*** | ***SEE*** | | ***df*** | ***AdjR^2^*** | |  | | **PAL** | 982.13 | 162.02 | 36.74 | <0.001 | 2.25 |
|  |  | **PAL** | 1303.35 | 115.50 | 127.35 | <0.001 | 1.51 |  | 24 | 180.35 | | 23 | 83.97 | |  |  | ***model*** | ***N*** | ***SEE*** | ***df*** | ***AdjR^2^*** |  |
|  |  | ***model*** | ***N*** | ***SEE*** | ***df*** | ***AdjR^2^*** |  |  | | | | | | | | |  | 29 | 179.84 | 28 | 87.98 |  |
|  |  |  | 53 | 190.10 | 52 | 86.45 |  |  |  |  |  |  |  |  |  |  |  | | | | |  |
|  | Pontzer2 | **Factors** | **β** | **Std. err.** | **f** | **p** | **V.I.F.** | **Factors** | **β** | **Std. err.** | | **f** | **p** | | **V.I.F.** | | **Factors** | **β** | **Std. err.** | **f** | **p** | **V.I.F.** |
|  |  | **Intercept** | -2894.63 | 242.49 | 68.46 | <0.001 |  | **Intercept** | -2857.21 | 476.73 | | 31.17 | <0.001 | |  |  | **Intercept** | -2101.1 | 249.32 | 61.02 | <0.001 |  |
|  |  | **%FM** | 20.27 | 4.87 | 17.30 | <0.001 | 1.14 | **%FM** | 14.96 | 7.19 | | 4.33 | 0.049 | | 1.18 | | **VM CPM** | 0.87 | 0.26 | 11.57 | 0.002 | 2.24 |
|  |  | **VM CPM** | 0.42 | 0.16 | 6.84 | 0.012 | 1.56 | **PAL** | 1515.11 | 193.68 | | 61.20 | <0.001 | | 1.18 | | **PAL** | 858.11 | 191.45 | 20.09 | <0.001 | 2.24 |
|  |  | **PAL** | 1295.28 | 121.26 | 114.11 | <0.001 | 1.43 | ***model*** | ***N*** | ***SEE*** | | ***df*** | ***AdjR^2^*** | |  | | ***model*** | ***N*** | ***SEE*** | ***df*** | ***AdjR^2^*** |  |
|  |  | ***model*** | ***N*** | ***SEE*** | ***df*** | ***AdjR^2^*** |  |  | 24 | 197.44 | | 23 | 72.40 | |  |  |  | 29 | 212.52 | 28 | 81.09 |  |
|  |  |  | 53 | 205.35 | 52 | 79.56 |  |  | | | | | | | | |  | | | | |  |
|  | RMR + ACC PAEE | **Factors** | **β** | **Std. err.** | **f** | **p** | **V.I.F.** | **Factors** | **β** | **Std. err.** | | **f** | **p** | | **V.I.F.** | | **Factors** | **β** | **Std. err.** | **f** | **p** | **V.I.F.** |
|  |  | **Intercept** | -1616.96 | 102.94 | 265.46 | <0.001 |  | **Intercept** | -1810.99 | 165.68 | | 157.07 | <0.001 | |  |  | **Intercept** | -1424.85 | 139.82 | 89.30 | <0.001 |  |
|  |  | **VM CPM** | -0.56 | 0.08 | 48.86 | <0.001 | 1.43 | **VM CPM** | -0.52 | 0.09 | | 32.09 | <0.001 | | 1.06 | | **Age** | -5.35 | 2.22 | 5.82 | 0.024 | 1.11 |
|  |  | **PAL** | 1408.83 | 63.46 | 492.82 | <0.001 | 1.43 | **PAL** | 1492.7 | 84.49 | | 312.16 | <0.001 | | 1.06 | | **VM CPM** | -0.49 | 0.14 | 12.83 | 0.001 | 2.32 |
|  |  | ***model*** | ***N*** | ***SEE*** | ***df*** | ***AdjR^2^*** |  | ***model*** | ***N*** | ***SEE*** | | ***df*** | ***AdjR^2^*** | |  | | **PAL** | 1376.62 | 101.20 | 185.05 | <0.001 | 2.25 |
|  |  |  | 53 | 107.49 | 52 | 91.05 |  |  | 24 | 90.77 | | 23 | 93.14 | |  |  | ***model*** | ***N*** | ***SEE*** | ***df*** | ***AdjR^2^*** |  |
|  |  |  | | | | | |  | | | | | | | | |  | 29 | 122.21 | 28 | 90.44 |  |
|  |  |  |  |  |  |  |  |  |  |  |  |  |  |  |  |  |  |  |  |  |  |  |
|  | Vinken1 | **Factors** | **β** | **Std. err.** | **f** | **p** | **V.I.F.** | **Factors** | **β** | **Std. err.** | | **f** | **p** | | **V.I.F.** | | **Factors** | **β** | **Std. err.** | **f** | **p** | **V.I.F.** |
|  |  | **Intercept** | -3650.61 | 317.57 | 67.55 | <0.001 |  | **Intercept** | -4124.59 | 456.15 | | 40.71 | <0.001 | |  |  | **Intercept** | -2785.99 | 245.27 | 70.02 | <0.001 |  |
|  |  | **Sex** | 114.12 | 45.52 | 6.28 | 0.016 | 2.96 | **Age** | 9.33 | 4.22 | | 4.89 | 0.039 | | 1.00 | | **Age** | 11.65 | 3.89 | 8.99 | 0.006 | 1.11 |
|  |  | **Age** | 11.41 | 2.85 | 16.07 | <0.001 | 1.04 | **FFM** | 24.58 | 8.32 | | 8.73 | 0.008 | | 1.07 | | **VM CPM** | 0.90 | 0.24 | 14.02 | 0.001 | 2.32 |
|  |  | **FFM** | 11.60 | 4.77 | 5.92 | 0.019 | 3.00 | **PAL** | 1563.22 | 169.65 | | 84.91 | <0.001 | | 1.07 | | **PAL** | 994.26 | 177.52 | 31.37 | <0.001 | 2.25 |
|  |  | **VM CPM** | 0.51 | 0.15 | 12.31 | 0.001 | 1.52 | ***model*** | ***N*** | ***SEE*** | | ***df*** | ***AdjR^2^*** | |  | | ***model*** | ***N*** | ***SEE*** | ***df*** | ***AdjR^2^*** |  |
|  |  | **PAL** | 1315.37 | 117.20 | 125.97 | <0.001 | 1.53 |  | 24 | 181.41 | | 23 | 83.82 | |  |  |  | 29 | 196.83 | 28 | 88.09 |  |
|  |  | ***model*** | ***N*** | ***SEE*** | ***df*** | ***AdjR^2^*** |  |  | | | |  | | |  |  |  | |  | |  |  |
|  |  |  | 53 | 191.64 | 52 | 86.48 |  |  |  |  |  |  |  |  |  |  |  |  |  | |  |  |
|  | Vinken2 | **Factors** | **β** | **Std. err.** | **f** | **p** | **V.I.F.** | **Factors** | **β** | **Std. err.** | | **f** | **p** | | **V.I.F.** | | **Factors** | **β** | **Std. err.** | **f** | **p** | **V.I.F.** |
|  |  | **Intercept** | -1643.34 | 122.23 | 251.25 | <0.001 |  | **Intercept** | -373.66 | 199.35 | | 186.03 | <0.001 | |  |  | **Intercept** | -1477.39 | 146.15 | 187.47 | <0.001 |  |
|  |  | **BM** | 14.11 | 2.35 | 35.89 | <0.001 | 5.04 | **%FFM** | -17.06 | 2.54 | | 45.13 | <0.001 | | 1.15 | | **Age** | -3.99 | 1.72 | 5.35 | 0.029 | 1.08 |
|  |  | **FFM** | -13.62 | 2.69 | 25.55 | <0.001 | 5.04 | **PAL** | 1482.09 | 76.85 | | 371.94 | <0.001 | | 1.15 | | **%FM** | 11.30 | 3.76 | 9.03 | 0.006 | 1.11 |
|  |  | **VM CPM** | -0.22 | 0.06 | 11.43 | 0.001 | 1.57 | ***model*** | ***N*** | ***SEE*** | | ***df*** | ***AdjR^2^*** | |  | | **PAL** | 1337.87 | 58.26 | 527.42 | <0.001 | 1.19 |
|  |  | **PAL** | 1441.2 | 50.98 | 799.15 | <0.001 | 1.53 |  | 24 | 79.27 | | 23 | 94.15 | |  |  | ***model*** | ***N*** | ***SEE*** | ***df*** | ***AdjR^2^*** |  |
|  |  | ***model*** | ***N*** | ***SEE*** | ***df*** | ***AdjR^2^*** |  |  |  | | | | | |  |  |  | 29 | 88.76 | 28 | 95.23 |  |
|  |  |  | 53 | 83.47 | 52 | 95.06 |  |  |  |  |  |  |  |  |  |  |  | | | | | |
|  | Vinken3 | **Factors** | **β** | **Std. err.** | **f** | **p** | **V.I.F.** | **Factors** | **β** | **Std. err.** | | **f** | **p** | | **V.I.F.** | | **Factors** | **β** | **Std. err.** | **f** | **p** | **V.I.F.** |
|  |  | **Intercept** | -1421.36 | 303.58 | 61.09 | <0.001 |  | **Intercept** | -3973.76 | 1139.69 | | 53.59 | <0.001 | |  |  | **Intercept** | -1146.27 | 405.12 | 46.08 | <0.001 |  |
|  |  | **Sex** | 149.96 | 45.07 | 11.07 | 0.002 | 2.96 | **Height** | 16.87 | 7.15 | | 5.56 | 0.028 | | 1.07 | | **FFM** | 11.71 | 5.46 | 4.60 | 0.041 | 1.00 |
|  |  | **FFM** | 14.30 | 4.66 | 9.40 | 0.004 | 2.92 | **PAL** | 1486.73 | 162.81 | | 83.39 | <0.001 | | 1.07 | | **PAL** | 1053.87 | 111.28 | 89.69 | <0.001 | 1.00 |
|  |  | **PAL** | 1196.17 | 95.07 | 158.32 | <0.001 | 1.03 | ***model*** | ***N*** | ***SEE*** | | ***df*** | ***AdjR^2^*** | |  | | ***model*** | ***N*** | ***SEE*** | ***df*** | ***AdjR^2^*** |  |
|  |  | ***model*** | ***N*** | ***SEE*** | ***df*** | ***AdjR^2^*** |  |  | 24 | 174.26 | | 23 | 82.06 | |  |  |  | 29 | 184.73 | 28 | 76.30 |  |
|  |  |  | 53 | 189.83 | 52 | 77.61 |  |  | | | | | | |  |  |  | | | | |  |

|  |  | ♀&♂ | | | | | | ♀ | | | | | | ♂ | | | | | |
| --- | --- | --- | --- | --- | --- | --- | --- | --- | --- | --- | --- | --- | --- | --- | --- | --- | --- | --- | --- |
| **Equations’bias (PAL≤1.89)** | Plucker3 | **Factors** | **β** | **Std. err.** | **f** | **p** | **V.I.F.** | **Factors** | **β** | **Std. err.** | **f** | **p** | **V.I.F.** | **Factors** | **β** | **Std. err.** | **f** | **p** | **V.I.F.** |
|  |  | **Intercept** | -4447.8 | 274.65 | 196.01 | <0.001 |  | **Intercept** | -3498.6 | 428.26 | 26.06 | <0.001 |  | **Intercept** | -6655.90 | 598.01 | 254.00 | <0.001 |  |
|  |  | **Sex** | 266.96 | 22.09 | 146.05 | <0.001 | 1.59 | **BM** | 17.95 | 5.49 | 10.69 | 0.011 | 3.26 | **Age** | 13.53 | 1.40 | 93.82 | <0.001 | 1.53 |
|  |  | **Age** | 10.20 | 1.79 | 32.45 | <0.001 | 1.12 | **FM** | -20.21 | 7.28 | 7.70 | 0.024 | 3.27 | **Height** | 16.41 | 3.86 | 18.11 | 0.002 | 4.06 |
|  |  | **%FFM** | 7.90 | 2.79 | 8.01 | 0.010 | 1.46 | **PAL** | 1676.86 | 217.32 | 59.54 | <0.001 | 1.02 | **FFM** | -11.89 | 3.18 | 13.93 | 0.005 | 3.39 |
|  |  | **PAL** | 1966.79 | 108.65 | 327.71 | <0.001 | 1.17 | ***model*** | **N** | **SEE** | **df** | **AdjR^2^** |  | **FM** | -7.58 | 2.95 | 6.61 | 0.030 | 1.51 |
|  |  | ***model*** | **N** | **SEE** | **df** | **AdjR^2^** |  |  | 12 | 93.09 | 11 | 83.24 |  | **VM CPM** | -0.42 | 0.12 | 11.43 | 0.008 | 3.31 |
|  |  |  | 28 | 91.89 | 27 | 96.65 |  |  | | | | |  | **PAL** | 2331.74 | 113.88 | 419.23 | <0.001 | 3.49 |
|  |  |  | | | | |  |  |  |  |  |  |  | ***model*** | **N** | **SEE** | **df** | **AdjR^2^** |  |
|  |  |  |  |  |  |  |  |  |  |  |  |  |  |  | 16 | 47.15 | 15 | 99.02 |  |
|  | Pontzer2 | **Factors** | **β** | **Std. err.** | **f** | **p** | **V.I.F.** | **Factors** | **β** | **Std. err.** | **f** | **p** | **V.I.F.** | **Factors** | **β** | **Std. err.** | **f** | **p** | **V.I.F.** |
|  |  | **Intercept** | -2215.74 | 471.43 | 18.55 | <0.001 |  | **Intercept** | -3252.19 | 548.96 | 40.23 | <0.001 |  | **Intercept** | -1191.02 | 232.88 | 23.80 | <0.001 |  |
|  |  | **BM** | -8.74 | 3.67 | 5.68 | 0.025 | 1.32 | **PAL** | 1986.50 | 313.19 | - | - | - | **VM CPM** | 1.57 | 0.32 | - | - | - |
|  |  | **FM** | 18.56 | 7.72 | 5.78 | 0.024 | 1.30 | ***model*** | **N** | **SEE** | **df** | **AdjR^2^** |  | ***model*** | **N** | **SEE** | **df** | **AdjR^2^** |  |
|  |  | **PAL** | 1517.88 | 229.54 | 43.73 | <0.001 | 1.01 |  | 12 | 135.50 | 11 | 78.10 |  |  | 16 | 223.59 | 15 | 60.32 |  |
|  |  | ***model*** | **N** | **SEE** | **df** | **AdjR^2^** |  |  | | | | |  |  | | | | |  |
|  |  |  | 28 | 208.03 | 27 | 66.10 |  |  |  |  |  |  |  |  |  |  |  |  |  |
|  | Vinken1 | **Factors** | **β** | **Std. err.** | **f** | **p** | **V.I.F.** | **Factors** | **β** | **Std. err.** | **f** | **p** | **V.I.F.** | **Factors** | **β** | **Std. err.** | **f** | **p** | **V.I.F.** |
|  |  | **Intercept** | -4306.39 | 625.95 | 24.77 | <0.001 |  | **Intercept** | -3705.28 | 704.24 | 14.36 | 0.001 |  | **Intercept** | -1686.14 | 256.63 | 40.57 | <0.001 |  |
|  |  | **Age** | 13.22 | 4.15 | 10.13 | 0.004 | 1.07 | **Age** | 19.56 | 5.35 | 13.36 | 0.006 | 1.32 | **VM CPM** | 2.26 | 0.35 | - | - | - |
|  |  | **%FFM** | 11.45 | 5.52 | 4.29 | 0.049 | 1.01 | **%FM** | -22.44 | 7.20 | 9.71 | 0.014 | 1.30 | ***model*** | **N** | **SEE** | **df** | **AdjR^2^** |  |
|  |  | **PAL** | 1707.49 | 247.53 | 47.58 | <0.001 | 1.08 | **PAL** | 2091.58 | 375.28 | 31.06 | <0.001 | 1.02 |  | 16 | 246.40 | 15 | 72.51 |  |
|  |  | ***model*** | **N** | **SEE** | **df** | **AdjR^2^** |  | ***model*** | **N** | **SEE** | **df** | **AdjR^2^** |  |  | | | | |  |
|  |  |  | 28 | 218.52 | 27 | 72.53 |  |  | 12 | 160.69 | 11 | 78.46 |  |  |  |  |  |  |  |

Table S3. General Linear Models with backward deletion applied to our entire sample, to individuals with physical activity levels (PAL) ≤1.89, and separated by sex using age, body mass (BM), height, fat-free mass (FFM), fat mass (FM), percentage of fat-free mass (%FFM), and percentage of fat mass (%FM), vector magnitude counts per minute per day (VM CPM) and physical activity level (PAL), as predictors of predictive equations bias. When sex is significant in the models 1 = female, -1 = male. Factors were eliminated from the models when multicollinearity was detected. ♀= Females; ♂= Males. V.I.F.: variance inflation factor. # sample size reduced by three females in the models to meet accelerometry criteria for valid days.

| **Whole sample** | ♀&♂  (n=56) | **Factors** | **β** | **Std.err.** | **f** | **p** | **V.I.F.** |
| --- | --- | --- | --- | --- | --- | --- | --- |
|  |  | **Intercept** | 389.29 | 355.90 | 28.54 | <0.001 |  |
|  |  | **BM** | 32.49 | 5.03 | 41.70 | <0.001 | 1.32 |
|  |  | **FM** | -31.19 | 4.52 | 7.01 | 0.011 | 1.46 |
|  |  | **VM CPM** | 1.26 | 0.23 | 29.94 | <0.001 | 1.13 |
|  |  | **Model** | **N** | **SEE** | **Df** | **AdjR^2^** |  |
|  |  |  | 53 # | 348.60 | 52 | 61.38 |  |
|  | ♀  (n=27) | **Factors** | **β** | **Std.err.** | **f** | **p** | **V.I.F.** |
|  |  | **Intercept** | -716.62 | 659.43 | 29.38 | <0.001 | - |
|  |  | **RMR** | 2.44 | 0.45 | - | - |  |
|  |  | **Model** | **N** | **SEE** | **Df** | **AdjR^2^** |  |
|  |  |  | 27 | 330.84 | 26 | 52.18 |  |
|  | ♂  (n=29) | **Factors** | **β** | **Std.err.** | **f** | **p** | **V.I.F.** |
|  |  | **Intercept** | -239.98 | 539.64 | 38.67 | <0.001 |  |
|  |  | **FFM** | 36.25 | 8.08 | 20.11 | <0.001 | 1.00 |
|  |  | **VM CPM** | 1.73 | 0.22 | 61.66 | <0.001 | 1.00 |
|  |  | **Model** | **N** | **SEE** | **Df** | **AdjR^2^** |  |
|  |  |  | 29 | 273.29 | 28 | 72.91 |  |

| **PAL**  **≤1.89** | ♀&♂  (n=28) | **Factors** | **β** | **Std.err.** | **f** | **p** | **V.I.F.** |
| --- | --- | --- | --- | --- | --- | --- | --- |
|  |  | **Intercept** | -383.25 | 402.24 | 34.43 | <0.001 |  |
|  |  | **VM CPM** | 1.20 | 0.28 | 18.73 | <0.001 | 1.00 |
|  |  | **RMR** | 1.41 | 0.20 | 51.28 | <0.001 | 1.00 |
|  |  | **Model** | **N** | **SEE** | **Df** | **AdjR^2^** |  |
|  |  |  | 25 # | 259.46 | 24 | 73.59 |  |
|  | ♀  (n=12) | **Factors** | **β** | **Std.err.** | **f** | **p** | **V.I.F.** |
|  |  | **Intercept** | -666.89 | 526.84 | 37.68 | <0.001 | - |
|  |  | **RMR** | 2.21 | 0.36 | - | - |  |
|  |  | **Model** | **N** | **SEE** | **Df** | **AdjR^2^** |  |
|  |  |  | 12 | 186.74 | 11 | 76.93 |  |
|  | ♂  (n=16) | **Factors** | **β** | **Std.err.** | **f** | **p** | **V.I.F.** |
|  |  | **Intercept** | 29.52 | 467.88 | 25.38 | <0.001 |  |
|  |  | **BM** | 25.06 | 5.47 | 21.00 | <0.001 | 1.00 |
|  |  | **VM CPM** | 1.68 | 0.32 | 27.96 | <0.001 | 1.00 |
|  |  | **Model** | **N** | **SEE** | **Df** | **AdjR^2^** |  |
|  |  |  | 16 | 220.64 | 15 | 76.48 |  |

Table S4. General Linear Models with backward deletion applied to our entire sample and by sex using age, body mass (BM), height, fat-free mass (FFM), fat mass (FM), percentage of fat-free mass (%FFM), and percentage of fat mass (%FM), resting metabolic rate (RMR) and vector magnitude counts per minute per day (VM CPM), as predictors of total energy expenditure. Factors were eliminated from the models when multicollinearity was detected. ♀= Females; ♂= Males. V.I.F.: variance inflation factor. # Sample size reduced in the model to meet accelerometry criteria for valid days.

**References:**

Plucker, A. *et al.* Adult energy requirements predicted from doubly labeled water. Int J Obes **42**, 1515–1523 (2018).

Pontzer, H. *et al.* Daily energy expenditure through the human life course. *Science (1979)* **373**, 808–812 (2021).

Vinken, A. G. *et al.* Equations for predicting the energy requirements of healthy adults aged 18–81 y. *Am J Clin Nutr* **69**, 920–926 (1999).
